# Variation in biochemical, physiological and ecophysiological traits among the teak (*Tectona grandis* Linn. f) seed sources of India

**DOI:** 10.1038/s41598-022-15878-0

**Published:** 2022-07-08

**Authors:** M. V. Jawahar Vishnu, K. T. Parthiban, M. Raveendran, S. Umesh Kanna, S. Radhakrishnan, Rubab Shabbir

**Affiliations:** 1 Forest College and Research Institute, Tamil Nadu Agricultural University, Mettupalayam, 641 301 India; 2 Tamil Nadu Agricultural University, Coimbatore, 641 003 India; 3 Department of Plant Breeding and Genetics, University of Agriculture, Faisalabad, 38040 Pakistan

**Keywords:** Biochemistry, Physiology, Plant sciences

## Abstract

Teak being an iconic timber species the studies on its physiological and biochemical traits are very limited in India and worldwide. As a result, the current study aimed to assess biochemical parameters such as chlorophyll a, chlorophyll b, total chlorophyll, carotenoids, chlorophyll ab ratio, proline content, and peroxidase activity, along with physiological parameters such as Chlorophyll stability index, relative water content, and leaf area, as well as ecophysiological traits such as net photosynthetic rate (Pn), stomatal conductance (Gs), intercellular CO_2_ concentration (Ci), transpiration rate (Tr), Leaf temperature, intrinsic water-use efficiency (iWUE), instantaneous water use efficiency and intrinsic carboxylation efficiency of thirty teak seed sources collected from different states of India. FCRITK 19, FCRITK 21, FCRITK 25, FCRITK 29, and FCRITK 05 were reported to have a greater photosynthetic rate (> 17 µmol m^−2^ s^−1^) coupled with a relative water content of more than 50% and a chlorophyll stability index of more than 60%, which could be used in a future genetic improvement programme. Correlation analysis indicated that water use efficiency was found to be strongly but negatively correlated with transpiration rate (−0.601) and stomatal conductance (−0.910). The proline content had a substantial positive correlation with the chlorophyll stability index (0.890), signifying that they are associated with abiotic stress conditions. Cluster analysis was attempted to discriminate the sources based on biochemical, physiological and ecophysiological traits. Eleven sources (FCRITK 25, FCRITK 27, FCRITK 29, FCRITK 14, FCRITK 30, FCRITK 16, FCRITK 05, FCRITK 13, FCRITK 02, FCRITK 17 and FCRITK 15) exhibited superior performance compared to rest of the sources.

## Introduction

Teak (*Tectona grandis* Linn. f) (Lamiaceae) is considered as the "Royal Timber" since it is one of the most important and iconic timber species in the world^1^. It is native to India, Myanmar, the Laos People’s Democratic Republic and Thailand, and naturalized in Java, Indonesia^2,3^. Teak provides premium timber with several desirable properties including high durability, strength and workability; resistance to fungi, termites and weathering; and a beautiful grain and colour^2,4^. Teak has multifarious uses including building, bridge and wharf construction, piles, furniture, cabinet work and railcars besides its utility in general carpentry works. Teak differs widely throughout India in terms of colour, grain, and texture, as well as physical, chemical, anatomical, and mechanical properties^5,6^. For structural demands such as shipbuilding and construction, trees from the Western Ghats region with considerable rainfall are favoured. Teak from Central India is preferred for furniture and cabinet manufacturing because of its hue (golden yellow, pink coloured heartwood), texture, ornamental figure, and attractive grain^7^. Teak's heartwood qualities are primarily determined by wood extractives, which are regulated by genetic and environmental influences^8^.

Since the early 1970s, there has been an increase in teak plantations due to rising global demand for teak wood and a significant decline in currently accessible resources^9,10^. Choosing the best teak origins is a basic requirement for maximizing productivity, especially because teak yields and quality vary a lot depending on planting site conditions^4,10^. The photosynthetic features are the key quantifiable indicators of plant growth. Information on variation in photosynthetic parameters and their relationship with growth traits helps us understand underlying processes and responses, and will be useful in tree improvement programs^11^.

Plant growth and survival will be harmed more severely and frequently as a result of climate change. The ability of a plant to cope with stress and recuperate determines its ability to modify growth and development under harsh situations. Growing periods with water scarcity can lead to decreased rates of height and diameter growth, reduced resistance to biotic and abiotic factors and changes in the timing and rate of physiological processes^12^. Teak plantations, like many other tropical plant species, are subjected to sustained periods of drought stress. As a result, it was expected that when plants were exposed to drought stress, a significant number of simultaneous changes in morphological, physiological, and biochemical responses would occur and that these changes would improve the plant's ability to survive and proliferate during drought times^13^. Teak being a widely adopted species the reports on its physiological and biochemical studies are very limited in India and worldwide. In 2013, the United Nations Climate Change Conference (UNFCCC) adopted seven agreements on forests, reiterating the importance of forests in reducing greenhouse gas emissions from industry. Carbon sequestration in terrestrial ecosystems can help humans adapt to present and future environmental changes by reducing the pace at which greenhouse gases accumulate in the atmosphere^14^. It is found that Teak plantations have a high potential for carbon sequestration, depending on the species, plantation techniques, and agronomical approaches used in maintenance and aftercare. Teak plants also aid in climate change mitigation by absorbing a variety of green gases, converting and fixing them into biomass, and returning oxygen to the environment. Aside from that, they have a lot of potential for enriching the forest floor to help with growth and biomass^15^.

Briggs and Shantz^16^ created the concept of water use efficiency (WUE) 100 years ago, demonstrating a link between plant yield and water use. They used the phrase "water use efficiency" to describe how much biomass a plant produces per unit of water consumed. The ability of trees to exploit water and nutrient resources plays a significant role in their adaptability to environmental changes. The ratio of net photosynthetic CO_2_ assimilation (A) to stomatal conductance (gs) is used to calculate tree-level intrinsic water-use efficiency (iWUE). In terrestrial ecosystems, iWUE is a crucial component of water-carbon coupling and process management, as well as a mechanism for trees to adapt to changing conditions^17^. The iWUE of teak seed sources could aid in improving drought tolerance in teak during advanced generations breeding programmes. Despite this, no attempt has been made to evaluate the relationship between WUE and other quantitative variables that affect species growth and most of the previous studies focus only on teak sources from limited areas. Against this backdrop, the objective of this study is to screen teak sources for high photosynthetic efficiency based on ecophysiological and biochemical parameters, as well as to understand the role of iWUE in tree growth and to identify the best sources for plantation development in dry areas and future genetic improvement work.

## Materials and methods

The experiment was conducted in one year old seed source evaluation trial established at Forest College and Research Institute, Mettupalayam (11$^\circ$ 19′ N; 76$^\circ$ 56′ E; 300 m above MSL) during 2021. The materials for the present study consisted of 30 teak seed sources (Table 1) collected from selected plus trees of eleven different states viz*.,* Tamil Nadu, Tripura, Maharashtra, Odisha, Gujarat, Kerala, Karnataka, Andhra Pradesh, Madhya Pradesh, Chhattisgarh and Jharkhand with the help of respective forest department officials along with proper permissions. The assembled seed sources were established in the seed source evaluation trial using Randomized Block Design (RBD) with 8 plants per plot with three replications at an espacement of 4 m $\times$ 4 m. All analyses were performed following the relevant guidelines and regulations.

**Table 1:** Source details of thirty teak seed sources investigated in the study with their geographical locations.

S. No	Place	State	Latitude	Longitude	Assigned Number
1	Nellithurai	Tamil Nadu	11^o^ 17′ 03″ N	76^o^ 51′ 55″ E	FCRITK 01
2	Nellithurai	Tamil Nadu	11^o^ 17′ 01″ N	76^o^ 51′ 55″ E	FCRITK 02
3	Kallar	Tamil Nadu	11^o^ 20′ 20″ N	76^o^ 52′ 31″ E	FCRITK 03
4	Omapalayam	Tamil Nadu	11^o^ 30′35″ N	76^o^ 91′61″ E	FCRITK 04
5	Kallar	Tamil Nadu	11^o^ 20′23″ N	76^o^ 52′ 20″ E	FCRITK 05
6	Kallar RF	Tamil Nadu	11^o^ 20′24″ N	76^o^ 52′ 36″ E	FCRITK 06
7	Agartala	Tripura	23^o^ 83′ 15″ N	91^o^ 28′ 68″ E	FCRITK 07
8	Nellithurai	Tamil Nadu	11^o^ 16′ 56″ N	76^o^51′ 58″ E	FCRITK 08
9	Vilamarathur	Tamil Nadu	11^o^ 15′ 50″ N	76^o^ 50′ 52″ E	FCRITK 09
10	Salem	Tamil Nadu	11^o^ 66′ 43″ N	78^o^ 14′ 60″ E	FCRITK 10
11	Burliyar	Tamil Nadu	11^o^ 34′ 37″ N	76^o^ 84′ 04″ E	FCRITK 11
12	Chandrapur	Maharashtra	19^o^ 96′ 15″ N	79^o^ 29′ 61″ E	FCRITK 12
13	Chandrapur	Maharashtra	19^o^ 96′ 15″ N	79^o^ 29′61″ E	FCRITK 13
14	Chandrapur	Maharashtra	19^o^ 96′ 15″ N	79^o^ 29′ 61″ E	FCRITK 14
15	Chandrapur	Maharashtra	19^o^ 96′ 15″ N	79^o^ 29′ 61″ E	FCRITK 15
16	Chandrapur	Maharashtra	19^o^ 96′ 15″ N	79^o^ 29′ 61″ E	FCRITK 16
17	Chandrapur	Maharashtra	19^o^ 96′ 15″ N	79^o^ 29′ 61″ E	FCRITK 17
18	Tanjore	Tamil Nadu	10^o^ 78′ 70″ N	79^o^ 13′ 78″ E	FCRITK 18
19	Rairakhol	Odisha	21^o^ 04′ 12″ N	84^o^ 20′ 60″ E	FCRITK 19
20	Dang	Gujarat	20^o^ 82′ 54″ N	73^o^ 70′ 07″ E	FCRITK 20
21	Nilambur	Kerala	11^o^ 28′ 55″ N	76^o^ 23′ 86″ E	FCRITK 21
22	Parambikulam	Kerala	10^o^ 37′ 78″ N	76^o^ 76′ 42″ E	FCRITK 22
23	Thenmala	Kerala	8^o^ 96′ 32″ N	77^o^ 06′ 51″ E	FCRITK 23
24	Shivamogga	Karnataka	13^o^ 92′ 99″ N	75^o^56′ 81″ E	FCRITK 24
25	Valsad	Gujarat	20^o^ 59′ 92″ N	72^o^ 93′ 42″ E	FCRITK 25
26	Dandeli	Karnataka	15^o^ 23′ 61″ N	74^o^ 61′ 73″ E	FCRITK 26
27	Khandwa	Madhya Pradesh	21^o^ 83′ 14″ N	76^o^ 34′ 98″ E	FCRITK 27
28	Vizianagaram	Andra Pradesh	18^o^ 10′ 67″ N	83^o^ 39′ 56″ E	FCRITK 28
29	Raipur	Chhattisgarh	21^o^ 25′ 14″ N	81^o^ 62′ 96″ E	FCRITK 29
30	Ranchi	Jharkhand	23^o^ 34′ 41″ N	85^o^ 30′ 96″ E	FCRITK 30

### Experimental Material

(a) The seeds were collected from a group of phenotypically superior trees (plus trees) which were selected based on the comparison tree method (b) The collected seeds were sown in raised beds with a medium of red soil, sand and farm yard manure in the ratio of 2:1:1. The beds were watered at regular intervals and maintained for six months. After 6 months the stumps which are above 3–4 cm thickness at the collar region are selected and transplanted into polybags containing a medium of red soil, sand and farm yard manure (FYM) in the ratio of 2:1:1. After a month of transplantation the seedlings were planted in the main field (c) All the seeds were sown simultaneously within a timespan of a week (d) No chemical treatments or fertilizers were provided at the nursery stage. At the time of planting each seedling was supplemented with 250 g of FYM, 25 g of vermicompost and 5 g of DAP (Di Ammonium Phosphate). The following data were collected from one-year-old grown teak plants.

### Ecophysiological traits

The gas exchange parameters including net photosynthetic rate (Pn), stomatal conductance (Gs), intercellular CO_2_ concentration (Ci), transpiration rate (Tr) and Leaf temperature were measured using a Li 6400 photosynthetic system (Li-Cor, Inc., Nebraska, USA). From each replication, fully mature and expanded leaves of each source were measured during the period of 10:00 to 12:00 h in the morning.

The intrinsic water-use efficiency (iWUE) is defined as the ratio of net photosynthesis and stomatal conductance, expressed in the units of µmol mol^−1^^18^. Instantaneous water use efficiency was estimated as the ratio of net photosynthetic rate to transpiration rate^19^. The ratio of net photosynthetic rate to the intercellular CO_2_ concentration is termed as intrinsic carboxylation efficiency^20^.

### Determination of leaf area

The leaf area is estimated by the linear method as per Montgomery^21^ by using the following formula

$$ {\text{Leaf area }}\left( {{\text{cm}}^{{2}} } \right) \, = {\text{ K }}\left( {0.{836}} \right){\text{ x L x B}} $$

L = Maximum length of the leaf; B = Maximum breadth of the leaf; K = Leaf area constant.

The value of leaf area constant (K) was calculated as the ratio between actual leaf area and apparent leaf area. Apparent leaf area is calculated by multiplying the maximum length and breadth of the leaf.

### Determination of leaf water status

Physiologically functional leaves were collected and made into leaf discs of uniform size and the fresh weight, dry weight and turgid weight of the leaves were measured. The leaf relative water content (RWC) was calculated as per Barrs and Weatherly^22^: 100 X [(fresh weight − dry weight)/ (turgid weight − dry weight)].

### Biochemical parameters

Chlorophyll was extracted from fresh leaves with 80% acetone from 0.25 g leaves samples. The extract was measured spectrophotometrically at 475, 645 and 663 nm with a spectrophotometer respectively. Total chlorophyll and carotenoids contents were determined by standard methodology^23^. The chlorophyll stability index was estimated by following Murthy and Majumdar^24^.

Proline was determined following Bates^25^. In 10 mL of 3 percent sulfosalicylic acid, 1.0 g of leaf material was homogenized. The homogenate was centrifuged before being filtered using filter paper. The reaction mixture, which included 2 mL homogenate, 2 mL glacial acetic acid, and 2 mL ninhydrin reagent, was heated for 60 min and then cooled for 10 min on ice. 4 mL toluene was added to the reaction mixture and agitated well for 20–30 s. Using a spectrophotometer, the absorbance of the coloured solutions was measured at 520 nm.

$$ {\text{Proline }}\left( {\mu {\text{g}}/{\text{g}}} \right) \, = \, \left( {{\text{Absorbance of Sample }}*{\text{ K Value }}*{\text{Dilution Factor}}} \right) \, / \, \left[ {{\text{Weight of Sample }}\left( {\text{g}} \right)} \right] $$

where *K* represents concentration/absorbance.

Peroxidase assay was performed at 25C in 3 ml of 60 mm phosphate buffer (pH 6.1) containing 16 mM guaiacol and 2 mM H202. Increase in absorbance was recorded at 470 nm with a Unicam SP 1700 Spectrophotometer. The reaction was linear for 30 min. G6PDH activity was assayed in 1 ml 60 mM Tris–HCl (pH 8.1) buffer with 150 mM MgC2, 6 mM NADP, 20 mm glucose-6-P, and 50 µl enzyme preparation. Increase in NADPH absorbance was monitored at 340 nm. Peroxidase activity was estimated and expressed in min − 1 mg − 1 protein as described by Castillo^26^.

### Statistical analysis

One-way ANOVA was used to analyze the data of physiological and biochemical traits of different teak seed sources, and Duncan's multiple range test was used to compare treatment means. The IBM-SPSS analytical software programme version 20.0 (IBM Corporation, USA) was used to analyze the data. The clustering analysis was performed by the UPGMA method (Unweighted Pair Group Method with Arithmetic mean) employing Past 4.03 software^27^.

## Results

Variance analysis of biochemical parameters, physiological parameters and ecophysiological traits are presented in Table 2. There is a significant difference in the biochemical parameters, physiological parameters and ecophysiological traits except for leaf temperature.

**Table 2:** Variance analysis (ANOVA) of biochemical parameters, physiological parameters and ecophysiological traits among teak seed sources.

Category	Parameter	F	P
Biochemical traits	Chlorophyll a	21.15	< 0.0001**
	Chlorophyll b	43.25	< 0.0001**
	Total Chlorophyll	31.27	< 0.0001**
	Carotenoids	33.77	< 0.0001**
	Chlorophyll ab ratio	36.52	< 0.0001**
	Proline	81.92	< 0.0001**
	Peroxidase activity	23.79	< 0.0001**
Physiological traits	Relative water content	31.41	< 0.0001**
	Chlorophyll stability index	63.06	< 0.0001**
	Leaf area	63.18	< 0.0001**
Ecophysiological traits	Photosynthetic rate	67.918	< 0.0001**
	Transpiration rate	80.323	< 0.0001**
	Stomatal conductance	279.335	< 0.0001**
	Internal CO2	58.67	< 0.0001**
	Leaf temperature	0.216	1.000^ ns^
	Instantaneous water use efficiency	75.177	< 0.0001**
	Intrinsic water use efficiency	385.986	< 0.0001**
	Intrinsic carboxylation efficiency	73.363	< 0.0001**

**Highly significant difference at p < 0.001 level of probability, and ns—no significance.

Duncan’s multiple comparison analysis of the biochemical parameters varied significantly among different sources (p = 0.05) and are listed in Table 3. The ranges of the parameters like chlorophyll a ranged between 2.291 ± 0.09 (FCRITK 06) and 1.277 ± 0.05 (FCRITK 21) mg g^−1^, chlorophyll b varied from 1.244 ± 0.05 (FCRITK 19) to 0.406 ± 0.01 (FCRITK 21) mg g^−1^, total chlorophyll was between 3.449 ± 0.09 (FCRITK 06) and 1.739 ± 0.04 (FCRITK 21) mg g^−1^, carotenoids ranged between 1.070 ± 0.05 (FCRITK 26) to 0.516 ± 0.02 (FCRITK 18) mg g^−1^, chlorophyll ab ratio was between 3.680 ± 0.04 (FCRITK 17) and 1.396 ± 0.06 (FCRITK 19), proline content varied from 3.76 ± 0.07 (FCRITK 22) to 1.17 ± 0.05 (FCRITK 12) µg g^−1^ and peroxidase activity ranged from 0.048 ± 0.00 (FCRITK 22, FCRITK 27 and FCRITK 30) to 0.030 ± 0.00 (FCRITK 28) min^−1^ mg^−1^ respectively. These results advocated that different teak sources exhibited different biochemical characteristics.

**Table 3:** Values of biochemical parameters among teak seed sources from different states of India.

Sources	Chlorophyll a (mg g^−1^)	Chlorophyll b (mg g^−1^)	Total chlorophyll (mg g^−1^)	Carotenoids (mg g^−1^)	Chlorophyll ab ratio	Proline Content ( µg g ^−1^)	Peroxidase activity (min^−1^ mg^−1^)
FCRITK 01	1.695 ± 0.05 ijk	0.778 ± 0.02 hij	2.377 ± 0.12 mno	0.726 ± 0.00 jk	2.179 ± 0.03 ghij	2.67 ± 0.04 cdef	0.038 ± 0.00 fgh
FCRITK 02	2.075 ± 0.02 bcd	0.747 ± 0.02 ijk	3.391 ± 0.00 ab	0.884 ± 0.04 de	2.778 ± 0.09 c	3.62 ± 0.10 a	0.040 ± 0.00 defg
FCRITK 03	1.389 ± 0.04 mn	0.693 ± 0.01 jkl	2.232 ± 0.08 nop	0.633 ± 0.02 l	2.003 ± 0.08 ijkl	1.45 ± 0.07 jk	0.041 ± 0.00 def
FCRITK 04	2.140 ± 0.03 ab	0.920 ± 0.01 def	3.130 ± 0.15 cde	0.847 ± 0.01 defgh	2.325 ± 0.02 efgh	3.25 ± 0.15 b	0.046 ± 0.00 ab
FCRITK 05	2.075 ± 0.02 bcd	0.747 ± 0.01 ijk	3.391 ± 0.11 ab	0.884 ± 0.03 de	2.778 ± 0.10 c	1.24 ± 0.04 kl	0.034 ± 0.00 ijk
FCRITK 06	2.291 ± 0.09 a	0.974 ± 0.04 bcd	3.449 ± 0.09 a	0.884 ± 0.04 de	2.352 ± 0.08 efg	1.42 ± 0.03 kjl	0.042 ± 0.00 cde
FCRITK 07	1.913 ± 0.08 defg	0.785 ± 0.03 hij	2.609 ± 0.01 hijklm	0.865 ± 0.04 defg	2.436 ± 0.03 def	2.25 ± 0.08 h	0.037 ± 0.00 ghi
FCRITK 08	2.190 ± 0.09 ab	1.012 ± 0.05 bc	3.159 ± 0.15 bcd	0.978 ± 0.03 bc	2.165 ± 0.05 ghijk	2.72 ± 0.11 cdef	0.040 ± 0.00 defg
FCRITK 09	1.751 ± 0.03 ghij	1.020 ± 0.05 bc	2.725 ± 0.06 ghij	1.022 ± 0.03 ab	1.718 ± 0.02 m	1.28 ± 0.06 kl	0.035 ± 0.00 hij
FCRITK 10	1.893 ± 0.03 defg	0.749 ± 0.03 ijk	2.609 ± 0.10 hijklm	0.989 ± 0.02 b	2.528 ± 0.13 de	1.46 ± 0.03 jk	0.038 ± 0.00 fgh
FCRITK 11	1.859 ± 0.09 fghi	0.827 ± 0.02 ghi	2.696 ± 0.05 ghijk	0.743 ± 0.02 ijk	2.249 ± 0.07 fghi	2.35 ± 0.01 gh	0.039 ± 0.00 efg
FCRITK 12	1.550 ± 0.08 klm	0.765 ± 0.03 ij	2.493 ± 0.09 jklm	0.646 ± 0.02 l	2.026 ± 0.03 ijkl	1.17 ± 0.05 l	0.045 ± 0.00 abc
FCRITK 13	1.883 ± 0.09 efgh	0.730 ± 0.02 jk	2.696 ± 0.02 ghijk	0.724 ± 0.03 jk	2.578 ± 0.10 cde	1.65 ± 0.05 j	0.032 ± 0.00 jkl
FCRITK 14	1.841 ± 0.01 fghi	0.877 ± 0.04 efg	2.522 ± 0.10 ijklm	0.871 ± 0.03 def	2.099 ± 0.09 hijk	3.37 ± 0.12 b	0.043 ± 0.00 bcd
FCRITK 15	1.901 ± 0.07 defg	0.986 ± 0.02 bcd	2.870 ± 0.07 fgh	0.870 ± 0.02 def	1.927 ± 0.04 klm	1.89 ± 0.07 i	0.033 ± 0.00 jkl
FCRITK 16	2.063 ± 0.07 bcde	1.058 ± 0.02 b	3.043 ± 0.10 def	0.887 ± 0.03 de	1.949 ± 0.08 jklm	2.58 ± 0.05 efg	0.045 ± 0.00 abc
FCRITK 17	2.190 ± 0.06 ab	0.595 ± 0.01 mn	2.609 ± 0.04 hijklm	0.720 ± 0.01 jk	3.680 ± 0.04 a	2.82 ± 0.13 cde	0.034 ± 0.00 ijk
FCRITK 18	1.502 ± 0.05 lm	0.871 ± 0.03 efg	2.174 ± 0.10 opq	0.516 ± 0.02 m	1.724 ± 0.05 m	2.88 ± 0.08 c	0.037 ± 0.00 ghi
FCRITK 19	1.737 ± 0.03 ghij	1.244 ± 0.05 a	1.942 ± 0.06 qr	0.616 ± 0.02 l	1.396 ± 0.06 n	2.24 ± 0.07 h	0.032 ± 0.00 jkl
FCRITK 20	1.925 ± 0.08 defg	0.780 ± 0.04 hij	2.783 ± 0.02 ghi	0.795 ± 0.00 fghij	2.467 ± 0.10 def	1.42 ± 0.06 kjl	0.047 ± 0.00 ab
FCRITK 21	1.277 ± 0.05 n	0.406 ± 0.01 o	1.739 ± 0.04 r	0.609 ± 0.00 l	3.146 ± 0.14 b	2.25 ± 0.04 h	0.042 ± 0.00 cde
FCRITK 22	1.865 ± 0.01 fghi	0.671 ± 0.01 klm	2.551 ± 0.01 ijklm	0.872 ± 0.00 def	2.780 ± 0.11 c	3.76 ± 0.07 a	0.048 ± 0.00 a
FCRITK 23	1.493 ± 0.03 lm	0.633 ± 0.01 lmn	2.058 ± 0.06 pq	0.684 ± 0.01 kl	2.359 ± 0.05 efg	2.90 ± 0.04 c	0.031 ± 0.00 kl
FCRITK 24	2.051 ± 0.03 bcde	0.953 ± 0.02 cde	2.899 ± 0.05 efg	0.912 ± 0.00 cd	2.152 ± 0.08 ghijk	2.52 ± 0.01 fg	0.034 ± 0.00 hijk
FCRITK 25	1.834 ± 0.04 fghi	0.726 ± 0.03 jk	2.609 ± 0.13 hijklm	0.788 ± 0.02 hij	2.526 ± 0.09 de	2.24 ± 0.09 h	0.045 ± 0.00 abc
FCRITK 26	2.117 ± 0.02 abc	1.213 ± 0.01 a	3.362 ± 0.04 abc	1.070 ± 0.05 a	1.745 ± 0.00 m	2.86 ± 0.15 cd	0.046 ± 0.00 abc
FCRITK 27	1.700 ± 0.01 hijk	0.732 ± 0.03 jk	2.435 ± 0.04 klmn	0.790 ± 0.01 ghij	2.323 ± 0.03 efgh	2.67 ± 0.12 cdef	0.048 ± 0.00 a
FCRITK 28	1.463 ± 0.01 lm	0.578 ± 0.02 n	1.942 ± 0.00 qr	0.679 ± 0.01 kl	2.530 ± 0.08 de	2.89 ± 0.07 c	0.030 ± 0.00 l
FCRITK 29	1.943 ± 0.10 cdef	0.730 ± 0.01 jk	2.667 ± 0.12 ghijkl	0.813 ± 0.02 efghi	2.663 ± 0.14 cd	3.25 ± 0.03 b	0.035 ± 0.00 hijk
FCRITK 30	1.588 ± 0.07 jkl	0.861 ± 0.04 fgh	2.406 ± 0.07 lmno	1.051 ± 0.00 ab	1.844 ± 0.00 ml	2.60 ± 0.12 defg	0.048 ± 0.00 a
Mean	1.840 ± 0.03	0.822 ± 0.02	2.652 ± 0.05	0.812 ± 0.01	2.314 ± 0.05	2.39 ± 0.08	0.039 ± 0.00

Values followed by the different letter of each group were significantly different at p < 0.05 level of probability.

Among the teak sources the physiological parameters like leaf area varied from 3469.12 ± 120.49 (FCRITK 25) to 1292.32 ± 19.90 (FCRITK 06) cm^2^, relative water content ranged from 89.47 ± 2.37 (FCRITK 28) to 47.62 ± 1.75 (FCRITK 12) % and chlorophyll stability index differed from 92.14 ± 2.93 (FCRITK 22) to 38.54 ± 0.85 (FCRITK 20) % respectively (Table 4).

**Table 4:** Values of physiological parameters among teak seed sources from different states of India.

Sources	Chlorophyll stability index (%)	Relative water content (%)	Leaf Area (cm^2^)
FCRITK 01	82.93 ± 0.50 bcd	76.19 ± 2.96 defg	1655.09 ± 54.58 h
FCRITK 02	90.00 ± 4.49 a	88.24 ± 0.97 ab	2391.89 ± 8.82 de
FCRITK 03	49.35 ± 0.34 l	71.43 ± 3.17 fghi	1920.87 ± 23.18 g
FCRITK 04	88.89 ± 1.55 ab	62.50 ± 2.42 klm	1419.03 ± 25.69 i
FCRITK 05	58.97 ± 0.94 jk	50.00 ± 0.85 n	2457.84 ± 91.99 d
FCRITK 06	36.97 ± 0.44 m	61.11 ± 2.68 lm	1292.32 ± 19.90 i
FCRITK 07	64.44 ± 0.21 ij	85.71 ± 0.81 ab	2096.87 ± 99.12 fg
FCRITK 08	80.73 ± 1.51 cde	66.67 ± 2.90 hijkl	1915.58 ± 57.79 g
FCRITK 09	55.32 ± 0.31 k	68.42 ± 2.50 hijk	2185.49 ± 49.37 ef
FCRITK 10	47.78 ± 1.37 l	86.36 ± 2.39 ab	1971.04 ± 21.97 fg
FCRITK 11	67.74 ± 1.70 hi	70.59 ± 1.11 ghi	2026.09 ± 5.53 fg
FCRITK 12	56.98 ± 1.53 k	47.62 ± 1.75 n	1933.25 ± 17.93 g
FCRITK 13	40.86 ± 0.07 m	69.57 ± 1.53 ghij	2349.90 ± 55.13 de
FCRITK 14	86.21 ± 2.59 abc	77.78 ± 3.31 cdef	2818.95 ± 81.68 c
FCRITK 15	39.39 ± 1.22 m	77.27 ± 0.12 cdef	2561.23 ± 50.47 d
FCRITK 16	80.95 ± 2.25 cde	48.72 ± 1.57 n	2934.49 ± 50.13 c
FCRITK 17	81.11 ± 1.23 cde	65.00 ± 0.08 ijklm	2399.95 ± 123.85 d
FCRITK 18	81.33 ± 2.44 cde	63.16 ± 2.14 jklm	1481.58 ± 1.41 hi
FCRITK 19	73.13 ± 0.18 fgh	58.33 ± 1.60 m	1891.90 ± 89.16 g
FCRITK 20	38.54 ± 0.85 m	59.09 ± 0.22 m	2064.14 ± 27.86 fg
FCRITK 21	69.01 ± 1.91 ghi	61.11 ± 1.66 lm	1981.76 ± 68.32 fg
FCRITK 22	92.14 ± 2.93 a	72.22 ± 2.85 efgh	2017.80 ± 16.27 fg
FCRITK 23	77.00 ± 2.21 def	70.00 ± 1.99 ghi	1878.69 ± 56.70 g
FCRITK 24	68.18 ± 3.33 hi	78.95 ± 1.45 cde	1636.40 ± 71.91 h
FCRITK 25	64.13 ± 3.10 ij	82.35 ± 0.73 bcd	3469.12 ± 120.49 a
FCRITK 26	78.33 ± 1.21 def	78.26 ± 1.86 cde	1953.04 ± 16.19 g
FCRITK 27	67.78 ± 3.05 hi	83.33 ± 2.64 abc	3293.00 ± 166.96 ab
FCRITK 28	74.63 ± 2.32 efg	89.47 ± 2.37 a	1678.07 ± 41.72 h
FCRITK 29	86.90 ± 4.20 abc	60.00 ± 0.15 lm	3148.84 ± 133.66 b
FCRITK 30	80.72 ± 2.27 cde	77.78 ± 3.75 cdef	2838.29 ± 29.75 c
Mean	68.68 ± 1.76	70.24 ± 1.25	2188.80 ± 58.50

Values followed by the different letter of each group were significantly different at p < 0.05 level of probability.

Significant variations among the teak sources for ecophysiological traits is shown in Table 5. The net photosynthetic rate (Pn) varied from 18.65 ± 0.06 (FCRITK 19) to 13.16 ± 0.15 (FCRITK 06) µmol m^−2^ s^−1^, stomatal conductance (gs) differed between 0.87 ± 0.02 (FCRITK 08) and 0.14 ± 0.01 (FCRITK 22 and FCRITK 28) mol m^−2^ s^−1^, transpiration rate (E) ranged from 2.95 ± 0.03 (FCRITK 15) to 1.15 ± 0.06 (FCRITK 08) mmol m^−2^ s^–1^, intercellular CO_2_ concentration (Ci) was between 475.0 ± 15.23 (FCRITK 10) and 180.5 ± 6.49 (FCRITK 18) µl l^−1^ and leaf temperature ranged from 38.80 ± 0.08 (FCRITK 12) to 37.15 ± 0.50 (FCRITK 22) °C respectively.

**Table 5:** Values of ecophysiological traits among teak seed sources from different states of India.

Sources	Photosynthetic Rate (µmol m^−2^ s^−1^)	Transpiration Rate (mmol m^−2^ s^–1^)	Stomatal Conductance (mol m^−2^ s^−1^)	Intercellular CO_2_ Concentration (µl l^−1^)	Leaf Temperature (°C)	Instantaneous water use efficiency (µmol mmol^−1^)	Intrinsic water use efficiency (µmol mol^−1^)	Intrinsic carboxylation efficiency (µmol m^−2^ s^−1^(µl l^−1^)^−1^)
FCRITK 01	16.45 ± 0.29 ef	1.65 ± 0.05 efgh	0.15 ± 0.00 mn	359.5 ± 3.86 d	38.50 ± 1.70 a	9.97 ± 0.44 efg	109.67 ± 1.57 a	0.046 ± 0.00 ghi
FCRITK 02	16.65 ± 0.12 def	2.75 ± 0.05 abc	0.52 ± 0.01 e	345.5 ± 1.42 def	38.15 ± 0.83 a	6.05 ± 0.27 klm	32.02 ± 1.44 jk	0.048 ± 0.00 fgh
FCRITK 03	14.65 ± 0.01 klm	1.45 ± 0.02 hij	0.15 ± 0.00 mn	474.5 ± 3.77 a	38.60 ± 1.40 a	10.10 ± 0.41 def	97.67 ± 0.30 b	0.031 ± 0.00 p
FCRITK 04	14.12 ± 0.20 m	1.54 ± 0.07 ghi	0.16 ± 0.00 mn	251.5 ± 10.25 ij	37.80 ± 0.67 a	9.17 ± 0.05 fgh	88.25 ± 4.01 c	0.056 ± 0.00 cd
FCRITK 05	17.42 ± 0.10 bc	1.68 ± 0.04 efg	0.62 ± 0.02 d	363.5 ± 12.89 d	37.50 ± 0.43 a	10.37 ± 0.35 de	28.10 ± 1.23 kl	0.048 ± 0.00 fgh
FCRITK 06	13.16 ± 0.15 n	1.60 ± 0.08 fghi	0.48 ± 0.01 f	426.5 ± 20.36 b	38.65 ± 1.03 a	8.23 ± 0.11 hi	27.42 ± 0.56 kl	0.031 ± 0.00 p
FCRITK 07	13.25 ± 0.13 n	1.80 ± 0.04 ef	0.44 ± 0.01 g	345.0 ± 2.97 def	38.50 ± 1.15 a	7.36 ± 0.27 ij	30.11 ± 0.01 jkl	0.038 ± 0.00 lmn
FCRITK 08	14.35 ± 0.10 lm	1.15 ± 0.06 m	0.87 ± 0.02 a	461.0 ± 2.56 a	37.60 ± 1.82 a	12.48 ± 0.51 b	16.49 ± 0.82 o	0.031 ± 0.00 p
FCRITK 09	15.19 ± 0.19 ijk	2.55 ± 0.13 cd	0.68 ± 0.04 c	393.5 ± 2.31 c	38.50 ± 0.11 a	5.96 ± 0.08 klm	22.34 ± 0.65 m	0.039 ± 0.00 lmn
FCRITK 10	16.65 ± 0.12 def	1.65 ± 0.04 efgh	0.24 ± 0.01 jk	475.0 ± 15.23 a	38.35 ± 1.06 a	10.09 ± 0.41 def	69.38 ± 0.97 e	0.035 ± 0.00 no
FCRITK 11	15.05 ± 0.11 ijk	2.85 ± 0.12 ab	0.59 ± 0.00 d	422.5 ± 13.69 bc	38.45 ± 0.66 a	5.28 ± 0.27 m	25.51 ± 0.55 lm	0.036 ± 0.00 mno
FCRITK 12	16.89 ± 0.02 cde	1.23 ± 0.03 klm	0.19 ± 0.01 lm	410.5 ± 12.04 bc	38.80 ± 0.08 a	13.73 ± 0.63 a	88.89 ± 4.54 c	0.041 ± 0.00 jkl
FCRITK 13	15.56 ± 0.17 hi	2.75 ± 0.11 abc	0.51 ± 0.01 ef	313.5 ± 8.46 g	38.65 ± 0.47 a	5.66 ± 0.08 lm	30.51 ± 1.25 jkl	0.050 ± 0.00 fg
FCRITK 14	16.24 ± 0.30 fg	1.86 ± 0.07 e	0.36 ± 0.01 h	356.5 ± 1.89 de	37.55 ± 0.59 a	8.73 ± 0.31 h	45.11 ± 0.62 h	0.046 ± 0.00 ghi
FCRITK 15	14.95 ± 0.02 jk	2.95 ± 0.03 a	0.68 ± 0.03 c	424.5 ± 8.93 bc	38.25 ± 0.48 a	5.07 ± 0.11 m	21.99 ± 0.98 mn	0.035 ± 0.00 no
FCRITK 16	16.75 ± 0.26 def	1.85 ± 0.05 e	0.27 ± 0.01 ij	392.0 ± 8.95 c	37.45 ± 1.08 a	9.05 ± 0.15 gh	62.04 ± 2.13 f	0.043 ± 0.00 ijk
FCRITK 17	13.43 ± 0.12 n	1.63 ± 0.08 fghi	0.17 ± 0.00 mn	213.0 ± 1.14 k	37.55 ± 1.49 a	8.24 ± 0.31 hi	79.00 ± 0.18 d	0.063 ± 0.00 b
FCRITK 18	14.09 ± 0.18 m	2.63 ± 0.10 cd	0.81 ± 0.02 b	180.5 ± 6.49 l	38.35 ± 0.48 a	5.36 ± 0.13 m	17.40 ± 0.90 no	0.078 ± 0.00 a
FCRITK 19	18.65 ± 0.06 a	1.69 ± 0.07 efg	0.22 ± 0.00 kl	406.5 ± 17.96 bc	38.25 ± 0.50 a	11.04 ± 0.22 cd	84.77 ± 1.05 c	0.046 ± 0.00 ghi
FCRITK 20	17.15 ± 0.22 bcd	1.20 ± 0.06 lm	0.43 ± 0.02 g	297.0 ± 0.19 gh	38.30 ± 0.26 a	14.29 ± 0.42 a	39.88 ± 1.60 i	0.058 ± 0.00 c
FCRITK 21	18.39 ± 0.32 a	2.74 ± 0.13 abc	0.62 ± 0.02 d	362.0 ± 3.73 d	37.40 ± 1.63 a	6.71 ± 0.07 jk	29.66 ± 0.96 jkl	0.051 ± 0.00 ef
FCRITK 22	14.75 ± 0.23 kl	1.25 ± 0.01 jklm	0.14 ± 0.01 n	275.0 ± 6.73 hi	37.15 ± 0.50 a	11.80 ± 0.37 bc	105.36 ± 2.90 a	0.054 ± 0.00 de
FCRITK 23	15.80 ± 0.21 gh	1.50 ± 0.03 ghi	0.28 ± 0.00 i	419.5 ± 11.23 bc	37.25 ± 1.44 a	10.53 ± 0.41 de	56.43 ± 1.29 g	0.038 ± 0.00 lmn
FCRITK 24	14.05 ± 0.02 m	2.67 ± 0.00 bcd	0.54 ± 0.01 e	326.5 ± 9.56 efg	37.60 ± 1.95 a	5.26 ± 0.12 m	26.02 ± 0.15 lm	0.043 ± 0.00 ijk
FCRITK 25	18.25 ± 0.23 a	2.66 ± 0.03 bcd	0.62 ± 0.01 d	423.5 ± 12.65 bc	37.70 ± 1.14 a	6.86 ± 0.18 jk	29.44 ± 1.17 jkl	0.043 ± 0.00 ijk
FCRITK 26	16.45 ± 0.20 ef	2.49 ± 0.04 d	0.48 ± 0.02 f	413.5 ± 9.71 bc	37.90 ± 1.37 a	6.61 ± 0.26 jkl	34.27 ± 1.60 j	0.040 ± 0.00 klm
FCRITK 27	14.33 ± 0.22 lm	2.64 ± 0.10 bcd	0.23 ± 0.01 k	431.5 ± 15.65 b	37.65 ± 1.46 a	5.43 ± 0.14 m	62.30 ± 0.82 f	0.033 ± 0.00 op
FCRITK 28	15.45 ± 0.24 hij	1.42 ± 0.00 ijk	0.14 ± 0.01 n	345.5 ± 12.00 def	37.25 ± 0.74 a	10.88 ± 0.22 cde	110.36 ± 0.27 a	0.045 ± 0.00 hij
FCRITK 29	17.55 ± 0.31 b	1.39 ± 0.02 ijkl	0.16 ± 0.01 mn	321.5 ± 4.87 fg	37.80 ± 0.54 a	12.63 ± 0.49 b	109.69 ± 2.05 a	0.055 ± 0.00 cde
FCRITK 30	15.13 ± 0.10 ijk	1.79 ± 0.00 ef	0.38 ± 0.02 h	236.0 ± 11.42 jk	37.55 ± 1.29 a	8.45 ± 0.44 h	39.82 ± 1.50 i	0.064 ± 0.00 b
Mean	15.69 ± 0.16	1.97 ± 0.06	0.40 ± 0.02	362.2 ± 8.09	37.97 ± 0.17	8.71 ± 0.29	54.00 ± 3.34	0.045 ± 0.00

Values followed by the different letter of each group were significantly different at p < 0.05 level of probability.

Water use efficiency parameters like instantaneous water use efficiency, intrinsic water use efficiency and intrinsic carboxylation efficiency were found to be significant among the teak seed sources. Instantaneous water use efficiency ranged between 14.29 ± 0.42 (FCRITK 20) to 5.07 ± 0.11 (FCRITK 15) µmol mmol^−1^. Intrinsic water use efficiency varied from 110.36 ± 0.27 (FCRITK 28) to 16.49 ± 0.82 (FCRITK 08) µmol mol^−1^ and intrinsic carboxylation efficiency varied between 0.078 ± 0.00 (FCRITK 18) to 0.031 ± 0.00 (FCRITK 03, FCRITK 06 and FCRITK 08) µmol m^−2^ s^−1^(µl l^−1^)^−1^.

Correlation analysis (Table 6) of teak sources showed that the chlorophyll stability index had a positive significant correlation with proline content (0.890) whereas it had a significant negative correlation with leaf temperature (−0.580). Similarly proline content also had a significant negative correlation with leaf temperature (−0.588). Height and basal diameter revealed a substantial positive relationship (0.733). Stomatal conductance had a positive significant correlation with transpiration rate (0.553) and a significant negative correlation with water use efficiency (−0.910). Total chlorophyll (−0.371) and transpiration rate (−0.601) were found to have a negative significant correlation with water use efficiency.

**Table 6:** Correlation analysis among growth traits, biochemical parameters, physiological parameters and ecophysiological traits of teak seed sources.

	HT	BD	TC	CSI	PL	RWC	LA	PR	TR	SC	LT	WUE
HT	1.000	0.733**	0.193	0.129	0.008	0.034	−0.261	−0.210	0.030	0.179	0.153	−0.042
BD		1.000	0.261	−0.091	−0.121	−0.179	−0.241	−0.168	−0.075	0.018	0.376*	0.083
TC			1.000	−0.097	−0.054	−0.097	0.037	−0.202	0.055	0.298	0.114	−0.371*
CSI				1.000	0.890**	0.088	0.093	0.030	−0.131	−0.233	−0.580**	−0.318
PL					1.000	0.318	0.113	−0.110	0.000	−0.211	−0.588**	0.251
RWC						1.000	0.112	−0.239	0.324	−0.009	−0.076	−0.058
LA							1.000	0.332	0.231	−0.013	−0.291	−0.065
PR								1.000	0.012	−0.060	−0.100	0.131
TR									1.000	0.553**	0.132	−0.601**
SC										1.000	0.118	−0.910**
LT											1.000	−0.124
WUE												1.000

**Indicate highly significant difference at p < 0.01 level of probability.

*Significant difference at p < 0.05 level of probability.

HT—Height, BD—Basal Diameter, TC—Total Chlorophyll, CSI—Chlorophyll Stability Index, PL—Proline Content, RWC—Relative Water Content, LA—Leaf Area, PR—Net Photosynthetic Rate, TR—Transpiration Rate, SC—Stomatal Conductance, LT—Leaf Temperature and WUE—Intrinsic Water Use Efficiency.

**Figure 1:**
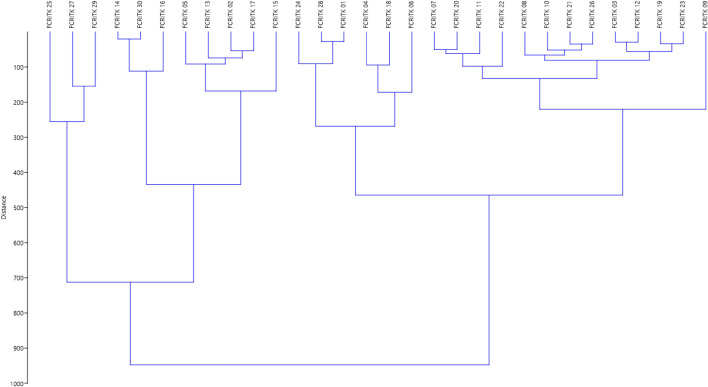
Cluster analysis on biochemical, physiological and ecophysiological traits among the teak seed sources.

The data on biochemical, physiological and ecophysiological traits were analyzed using hierarchical cluster analysis, by UPGMA method based on Euclidian distance for the thirty teak seed sources (Fig. 1). The sources were segregated into twelve clusters where the cluster XI had eight sources, clusters X and V had four sources each, cluster VII had three sources, clusters II, III and VIII had two sources each, whereas the remaining clusters have one source each.

## Discussion

The impacts of stress on photosynthetic physiology, as well as photosynthetic responses to light intensity and CO_2_ concentration, have been the focus of ecophysiological investigations on photosynthesis in forest trees till date^28^. The current study highlights the physiological and biochemical characteristics of teak seed sources from different locations. The objectives of this paper was to systematically measure photosynthetic gas exchange and chlorophyll parameters, correlate photosynthetic and biochemical characteristics with growth, and provide a strategy for rapid evaluation of teak seed sources in order to introduce, use, and improve teak resources in future breeding programmes.

### Biochemical parameters

Chlorophyll (Chl) is a key photosynthetic pigment in plants, influencing photosynthetic capacity and thus plant growth^29^. Changes in the amount of chlorophyll may also be a part of adaptive reactions. The current study implied that chlorophyll a, chlorophyll b, total chlorophyll and chlorophyll ab ratio registered significantly comparable values among the sources. The decreased values could be due to photoinhibition^30^. Chlorophyll loss is both a negative and adaptive component of stress since it reduces light absorption and hence the risk of further damage to the photosynthetic mechanism^31^. Higher values were noted in FCRITK 06, FCRITK 05, FCRITK 02 and FCRITK 26. Carotenoids play an important role in photosynthesis and photoprotection. They are well known for their antioxidant properties, in addition to their structural responsibilities. Carotenoids are also important for the formation of the light-harvesting complex and the non-radiative dissipation of surplus energy^31^. Carotenoids ranged from 1.070 ± 0.05 to 0.516 ± 0.02 mg g^−1^. Similar trends in chlorophylls and carotenoids were noticed in Teak^12,13,30,32,33^, Pungam^34^ and Rubber^35^. Many studies^36–38^ demonstrate that mild stress does not affect chlorophyll concentration.

Proline accumulation acts as an osmotic regulator and a protective mechanism in plants under abiotic stress^39^. Free proline is reported to induce stress tolerance in a variety of plants through dehydration of protoplasm. The amount of proline present in the teak clones was between the ranges of 3.76 ± 0.07 to 1.17 ± 0.05 µg g^−1^ which indicated that the plants can withstand abiotic stress conditions. The results are in corroboration with Teak^12,13,30^, Rubber^35^ and Populus^40^.

Peroxidase activity is an adaptive feature that helps to repair tissue metabolic damage by lowering the harmful quantities of H_2_O_2_ generated during cell metabolism. Peroxidase protects against oxidative stress by converting H_2_O_2_ to water and oxygen. The increase in peroxidase activity implies that the plant uses protective mechanisms (photo-protection) to cope with moisture stress. Increased peroxidase activity could suggest that the cell wall's mechanical characteristics have deteriorated^12^. The present study indicates that all the teak sources exhibited reduced peroxidase activity (0.048 ± 0.00 to 0.030 ± 0.00 min^−1^ mg^−1^) which might be due to reduced stress conditions. Similar trends have been noticed in teak^12,13^ and *Populus cathayana*^41^.

### Physiological parameters

Estimation of Relative Water Content is an appropriate method to assess the plant water status. RWC is also used as an index to screen the plants for drought tolerance. RWC of thirty teak sources ranged from 89.47 ± 2.37 to 47.62 ± 1.75%. All the teak sources barring FCRITK 12 and FCRITK 16 exhibited RWC of more than 50% indicating moderate to strong drought tolerance. The results are in corroboration with Teak^12,13,42^, Rubber^35^ and Populus^40^. Chlorophyll pigments are thermosensitive in nature and their degradation occurs when it is subjected to high temperature. Estimation of Chlorophyll Stability Index indicates the intensity of colour changes induced by heating. Since Chlorophyll Stability Index is a function of temperature, the property of chlorophyll pigments can be correlated with the drought tolerance of the plants. In the present study Teak sources FCRITK 22, FCRITK 02, FCRITK 04, FCRITK 29 and FCRITK 14 revealed more than 85% of Chlorophyll Stability Index which indicated high drought tolerance.

### Ecophysiological traits

The leaf is one of the most important organs in the plant system, and the plant's continuous development is dependent on its ability to persist. Physiologically, the leaf area represents the principal photosynthetic surface and supplies most of the photosynthates required by the plant components. As a result, estimating leaf area becomes an important aspect of growth analysis and is frequently used in physiological reasoning of agricultural productivity fluctuations. The leaf area of teak sources ranged from 3469.12 ± 120.49 to 1292.32 ± 19.90 cm^2^ which revealed that all the sources exhibited higher leaf area than other findings in teak^30,43^.

Photosynthesis is crucial for plant development and productivity. Plant photosynthesis is influenced not only by environmental conditions but also by the genetic traits of the plant. Photosynthetic activity is influenced by a complicated process of interaction between genetic and environmental factors in plants^44^. The photosynthetic rate was found to be higher in FCRITK 19, FCRITK 21, FCRITK 25, FCRITK 29 and FCRITK 05 indicating that these seed sources exhibit higher productivity in terms of biomass. Stomatal conductance was found to be in the higher range (0.87 to 0.14 mol m^−2^ s^−1^) which might be due to increased leaf temperature (38.80 to 37.15 °C) leading to increased photosynthesis^45^. Transpiration rate was found to be low among the teak seed sources which specifies that these sources can be utilized for plantation under drought-prone areas. Intercellular CO_2_ concentration ranged between 475.0 ± 15.23 to 180.5 ± 6.49 µl l^−1^ indicating that higher intercellular CO_2_ concentration associated with increased stomatal conductance affects the photosynthetic rate. Lower photosynthetic rate than the current investigation was documented in teak^11–13,33,42,46^, Similar noteworthy results were documented in *Pawlonia tomentosa*^47^, Eucalyptus species^48^, *Tabebuia aurea*^49^ and Rubber^35^.

The WUE of plants could be used as a key criterion for developing drought-tolerant varieties^50^. Instantaneous water use efficiency was found to be higher among teak sources in the current investigation, indicating that these sources are better at diverting water for photosynthesis than transpiration. The intrinsic water use efficiency varied significantly in the present study. Higher iWUE values (FCRITK 28, FCRITK 29, FCRITK01 and FCRITK 22) imply that these teak sources are better at carbon assimilation, resulting in higher productivity under drought stress. Other such findings in eucalyptus clones^51–53^ and teak^11,54^ lend support to the present investigation.

### Correlation analysis

Correlation analysis indicated that water use efficiency was significantly but negatively correlated with transpiration rate and stomatal conductance suggesting that the WUE may decrease when transpiration rate and stomatal conductance is high. Leaf temperature had a significant negative correlation with chlorophyll stability index and proline content. Chlorophyll stability index had a significant positive correlation with proline content indicating that they are directly related to abiotic stress conditions. Similar trends were noted in eucalyptus clones^53^. Significant correlations between growth traits and physiological parameters were observed in Salix species under normal conditions^55^ and in Teak under drought conditions^13^. Under drought stress conditions, positive correlations between chlorophyll content, growth traits, and physiological parameters were also found in other species such as *Alstonia macrophylla*, *Acacia auriculiformis*, *Artocarpus heterophyllus, Terminalia arjuna* and *Azadiracta indica*^56^.

### Cluster analysis

Cluster analysis was attempted to discriminate the sources based on biochemical, physiological and ecophysiological traits. It assisted in determining the most distant and closest sources for subsequent breeding. Eleven sources (FCRITK 25, FCRITK 27, FCRITK 29, FCRITK 14, FCRITK 30, FCRITK 16, FCRITK 05, FCRITK 13, FCRITK 02, FCRITK 17 and FCRITK 15) exhibited superior performance compared to rest of the sources. The results are in corroboration with other such findings of teak^12^.

## Conclusion

Plants are capable of adapting to abiotic stress and phenotypic plasticity in response to it. As a result, the ability of plants to respond to stress, as well as their ability to recover from stress and resume normal metabolism, is crucial. As a result, knowing biochemical, physiological, and ecophysiological features is crucial for tackling global climate change-related challenges, such as drought. In this study, teak seed sources FCRITK 19, FCRITK 21, FCRITK 25, FCRITK 29, and FCRITK 05 were discovered to have a higher photosynthetic rate, as well as a relative water content of more than 50% and a chlorophyll stability index of more than 60%, and could be used in a future genetic improvement programme. Continued research is essential on the correlation of these traits with genetic mechanisms, including the identification of potential genes linked to drought resistance. To improve the teak germplasm in India, greater research into the assessment of more specific features linked to growth, wood quality, and water-use efficiency in teak in multi-locational provenance experiments is essential.

## Supplementary Information

Supplementary Information.

## Data Availability

All data generated or analyzed during this study are included in this published article.
