# Author Correction: Variation in biochemical, physiological and ecophysiological traits among the teak (*Tectona grandis* Linn. f) seed sources of India

**DOI:** 10.1038/s41598-022-19038-2

**Published:** 2022-08-31

**Authors:** M. V. Jawahar Vishnu, K. T. Parthiban, M. Raveendran, S. Umesh Kanna, S. Radhakrishnan, Rubab Shabbir

**Affiliations:** 1 Forest College and Research Institute, Tamil Nadu Agricultural University, Mettupalayam, 641 301 India; 2 Tamil Nadu Agricultural University, Coimbatore, 641 003 India; 3 Department of Plant Breeding and Genetics, University of Agriculture, Faisalabad, 38040 Pakistan

Correction to: *Scientific Reports* https://doi.org/10.1038/s41598-022-15878-0, published online 08 July 2022

The original version of this Article contained an error in Affiliation 3, which was incorrectly given as ‘University of Agriculture, Faisalabad 38040, India.’ The correct affiliation is listed below:

Department of Plant Breeding and Genetics, University of Agriculture, Faisalabad 38040, Pakistan

The original Article has been corrected.

