# Supplementary material for: Variation in biochemical, physiological and ecophysiological traits among the teak (Tectona grandis Linn. f) seed sources of India
Source: Sci Rep. 2022 Jul 8;12:11677. doi: 10.1038/s41598-022-15878-0 (PMC9270387; doi:10.1038/s41598-022-15878-0)
Supplement: Supplementary file 1 — Supplementary Information. [file 41598_2022_15878_MOESM1_ESM.docx]

Supplementary Fig 1. Diagrammatic Depiction of Instantaneous water use efficiency among the teak sources

Supplementary Fig 2. Diagrammatic representation of intrinsic water use efficiency among the teak sources

Supplementary Fig 3. Graphical Depiction of Intrinsic carboxylation efficiency among the teak sources

Supplementary Fig 4. Graphical Depiction of Intercellular CO_2_ concentration among the teak sources

Supplementary Fig 5. Variation among the teak sources for Stomatal conductance

Supplementary Fig 6. Variation among the teak sources for transpiration conductance

Supplementary Fig 7. Variation among the teak sources for photosynthetic rate
